# Stress Tracker—Detecting Acute Stress From a Trackpad: Controlled Study

**DOI:** 10.2196/22743

**Published:** 2020-10-23

**Authors:** Rahul Goel, Michael An, Hugo Alayrangues, Amirhossein Koneshloo, Emmanuel Thierry Lincoln, Pablo Enrique Paredes

**Affiliations:** 1 Department of Radiology School of Medicine Stanford University Palo Alto, CA United States; 2 Department of Computer Science University of California Los Angeles Los Angeles, CA United States; 3 Department of Computer Science Institut supérieur d’électronique de Paris Paris France; 4 Department of Industrial, Manufacturing, and Systems Engineering Texas Tech University Lubbock, TX United States; 5 Department of Psychiatry and Behavioral Sciences School of Medicine Stanford University Palo Alto, CA United States

**Keywords:** precision health, well-being, trackpad, computer input device, computer interaction, stress sensing, affective interfaces, mental health

## Abstract

**Background:**

Stress is a risk factor associated with physiological and mental health problems. Unobtrusive, continuous stress sensing would enable precision health monitoring and proactive interventions, but current sensing methods are often inconvenient, expensive, or suffer from limited adherence. Prior work has shown the possibility to detect acute stress using biomechanical models derived from passive logging of computer input devices.

**Objective:**

Our objective is to detect acute stress from passive movement measurements of everyday interactions on a laptop trackpad: (1) *click*, (2) *steer*, and (3) *drag and drop*.

**Methods:**

We built upon previous work, detecting acute stress through the biomechanical analyses of canonical computer mouse interactions and extended it to study similar interactions with the trackpad. A total of 18 participants carried out 40 trials each of three different types of movement—(1) *click*, (2) *steer*, and (3) *drag and drop*—under both relaxed and stressed conditions.

**Results:**

The mean and SD of the contact area under the finger were higher when clicking trials were performed under stressed versus relaxed conditions (mean area: *P*=.009, effect size=0.76; SD area: *P*=.01, effect size=0.69). Further, our results show that as little as 4 clicks on a trackpad can be used to detect binary levels of acute stress (ie, whether it is present or not).

**Conclusions:**

We present evidence that scalable, inexpensive, and unobtrusive stress sensing can be done via repurposing passive monitoring of computer trackpad usage.

## Introduction

### Overview

Several health risks such as cardiovascular disease and immune deficiencies, which can diminish the quality of life and shorten life expectancy [1], are linked to repetitive daily acute stress (ie, short-term response to a perceived threat or challenge [2,3]). Stress can also have a profound impact on cognitive and emotional well-being. Advances in wearable technology enable affective and cognitive state measurements. Still, wearable devices can suffer from high attrition and low adoption, in general, and getting stable stress measurements “in the wild” (eg, heart rate variability [HRV] and electrodermal activity [EDA]) remains challenging.

Previous lab studies have shown that data from interactions with everyday devices, such as computer mouse movements, keyboard pressure, smartphone touch, and key swipes, or even the steering wheel of a car, can be used in an unobtrusive and scalable way to detect the presence of acute stress [4-8]. To the best of our knowledge, this paper presents the first work showing the feasibility of detecting acute stress from interactions with laptop computer trackpads.

There are several billion personal computing devices (PCDs) deployed in the world, and this number increases by around 300 million every year [9]. Laptops, or notebooks, represent the largest (42%) and the fastest-growing segment [9] among PCDs. Most laptop users prefer the trackpad over an external mouse, for improved mobility or due to space limitations. Investigating biomechanics and motor control of finger dynamics during trackpad use can lead to reliable detection of acute stress, especially for long-term unobtrusive continuous monitoring. We propose two research questions (RQs):

1. RQ1: Can we assess differences between stress versus relaxation through changes in the damping ratio (Γ) and damping frequency (ω) of a mass-spring-damper (MSD) model derived from finger strokes on a laptop trackpad, similar to our previous study on a computer mouse [4]?
2. RQ2: Are there differences between stress and relaxation in other metrics of finger dynamics, such as contact area, velocity, and acceleration, which are theoretically linked to muscle tension?

**Figure 1:**
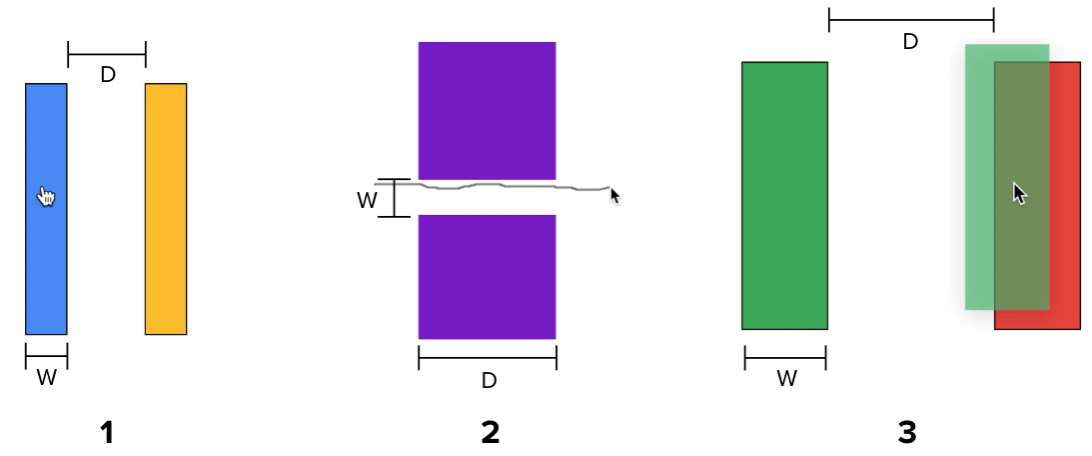
Computer tasks used in the study: (1) *click*, (2) *steer*, and (3) *drag and drop*. Different combinations of width (W) and distance (D) were randomly presented (see Table 1). In the click trials, subjects had to click anywhere on the blue rectangle first and then the yellow rectangle. In the *steer* trials, subjects had to hold down the left key and steer across the distance between the two rectangles. In the *drag-and-drop trials*, subjects had to click on the green rectangle, hold it, drag it, and then drop it over the red rectangle.

We conducted a within-subjects study (N=18) counterbalancing relaxed and stressed conditions. Subjects performed 40 trials each of three canonical computer tasks using a trackpad, namely, *clicking*, *steering,* and *drag and drop* (see Table 1 and Figure 1), similar to what Sun et al did with a computer mouse [4]. We focused our analyses on metrics derived from finger dynamics. Our results confirm a significantly higher mean and SD of the contact area under the finger for the tasks performed under stress compared to relaxed conditions, even among the initial 10% of the data (ie, 4 out of 40 *clicks*) from *clicking* trials. To the best of our knowledge, this is the first systematic study that links acute stress to finger strokes on the trackpad of a laptop device.

**Table 1:** Different task configurations by varying the distance and width parameters.

Task type	Distance by configuration, pixels					Width by configuration, pixels						
	1	2	3	4	5		1	2	3	4		
Click	64	128	256	512	1024		8	16	32	64		
Drag and drop	64	128	256	512	1024		16	32	64	128		
Steer	64	128	256	512	1024		8	16	32	64		

### Background

#### Stress Measurements

Stress can be measured via self-reports or physiological signals. Self-reported stress (SRS) can be measured through the Perceived Stress Scale (PSS) [10,11]. Typically, a simplified version with a single 11-item scale of stress, ranging from 0 to 10, is used in repeated-measure studies [4,12]. Although subjective responses are commonly used, they are not precise and suffer from response bias and noncompliance [13], making them impractical for long-term monitoring.

Complementarily, physiological measurement of stress can be done by an indirect observation of the autonomic nervous system (ANS) signal [14]. A popular metric is EDA [15,16]. EDA, formerly known as the galvanic skin response, is a measurement of skin conductance due to the activation of the eccrine sweat glands, which are only innervated by the sympathetic nervous system (SNS). The SNS is one of the branches of the ANS linked to the “fight or flight” response, the coordinated response by the body organs to a threat signal (ie, stressor). High average levels and an increase in skin conductance responses (SCRs) (ie, the number of EDA peaks) are associated with stress [16]. Another popular measure is HRV, which is the measurement of the time variation between R-R peaks of an electrocardiogram (ECG) signal due to the sinusoidal arrhythmia [14]. EDA and ECG can be obtained in ambulatory settings using wearable devices. However, these sensors are obtrusive, leading to dropout, and are also affected by motion artifacts. In this paper, we validated our stressor with SRS and EDA.

#### Links Between Stress, Motor Control, and Muscle Tension

**Figure 2:**
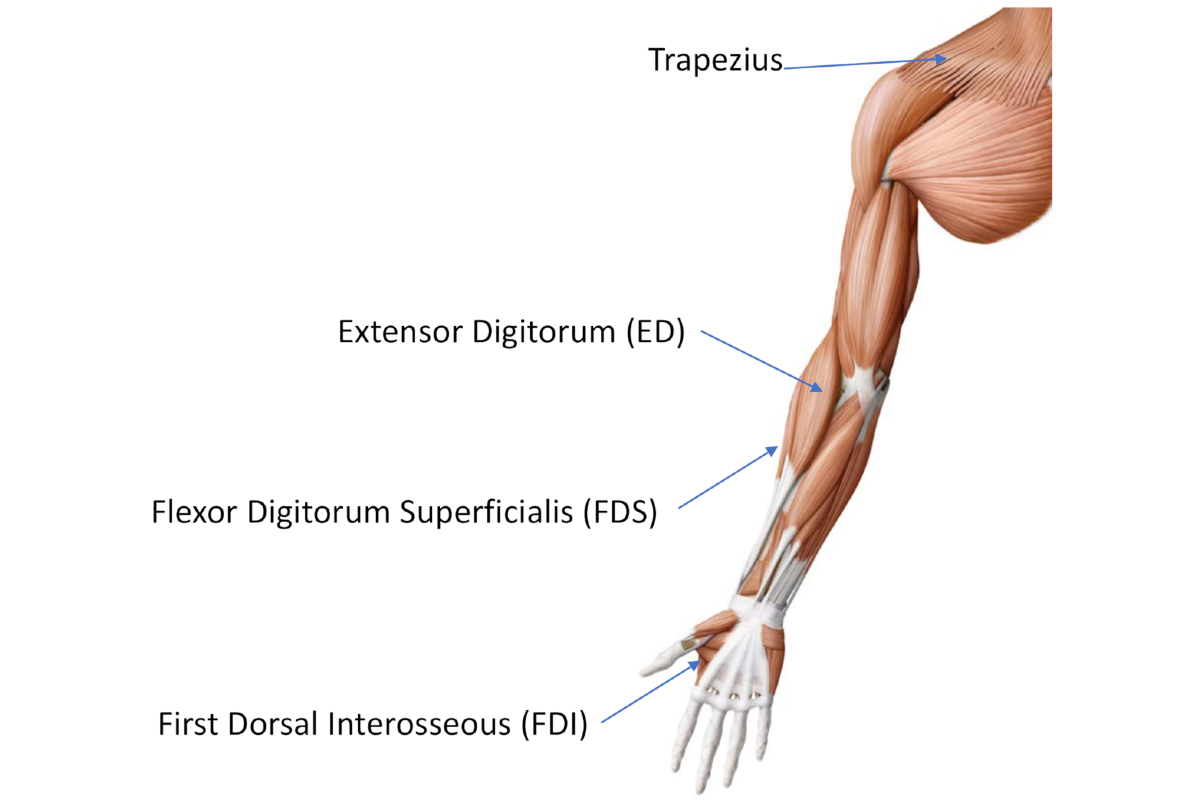
Main muscles activated when using computer input devices: mouse and trackpad.

Previous research suggests that hormones and neurotransmitters released during heightened stress arousal can influence motor output [17]. Noteboom et al [17] carried out a precision task that required a submaximal isometric pinch grip. Their research found that the steadiness in electromyography (EMG) activity in the first dorsal interosseous (FDI) muscle of the hand (see Figure 2), and the flexor digitorum superficialis muscle of the forearm, was increased during stress, especially in those with moderate anxiety trait compared to those with low anxiety trait. Laidlaw et al [18] measured the motor unit activity using intramuscular EMG recordings in the FDI during a pinch grip task. They concluded that increased variability of motor unit discharge is associated with reductions in steadiness. Coombes et al [19] showed that the force production in wrist and finger extensor muscles was increased during continuous exposure to unpleasant stimuli, compared to neutral stimuli, for a voluntary bimanual maximal isometric contraction task. Arnich et al [20] showed that nervous or anxious individuals have higher levels of movement variability while sitting (ie, a higher center of pressure dispersion on the seat). These studies provide evidence on how acute stress can influence variability while performing a motor task.

Additionally, various studies have shown that acute mental stress increases muscle tension in the neck, forehead, and arms [21-24]. The shoulder's trapezius muscle [25], as well as biceps and triceps [26], have shown direct changes due to mental arithmetic tasks. In this study, we used two types of biomechanical metrics: those that are influenced by changes in muscle tension and motor control variability due to stress.

#### Effects of Stress on the Manipulation of Computer Input Devices

Sun et al [4] showed a direct correlation between stress, muscle tension, and movement of a computer mouse by approximating the mouse movement trajectories as a step response of an MSD system. They calculated the Γ and ω, both of which were higher under stress. Hernandez et al showed a higher contact area with the surface of a capacitive mouse under stressful conditions compared to relaxed conditions [6]. Wahlstrom et al [27] showed that muscle activity in the right extensor digitorum (ED) and right trapezius muscles were greater in stressed situations evoked by time pressure and verbal provocation while working with a computer mouse. Carneiro et al [28] showed that acceleration and mean and maximum intensity (aka, pressure) of touch when interacting with touchscreen devices were higher under stressful conditions. While not the same as stress, Gao et al [7] showed that the velocity and contact area of the finger strokes during the *frustrated* state in comparison to the *relaxed* state were among the main features used in a machine learning (ML) prediction model of *frustration* during game-playing interactions on an iPod. To the best of our knowledge, there is no prior research on systematic stress detection using finger dynamics during laptop trackpad use.

#### Biomechanics of Trackpad Versus Mouse Usage

It is important to understand the biomechanical differences between trackpad and mouse use [29]. Direct comparisons between methods and metrics used with a computer mouse may not be feasible for a trackpad. Mouse use primarily triggers the large trapezius muscles for moving the entire arm leading to horizontal and vertical movement of the cursor on the screen [29]. Although both mouse and trackpad movements use the ED muscle for *clicking*, trackpad displacement mainly involves the rather small FDI hand muscle [29]. Figure 2 shows the relative sizes of the muscles of the arm and hand.

In comparison to the trackpad, mouse use induces larger shoulder abduction and smaller elbow flexion [29]. Trackpad use requires a more static posture of the upper arm to maintain stabilization of the hand but a more rigorous movement of fingers [29]. Stress detection via an approximation of muscle tension [4] benefits from more muscle activity. However, the absolute level of muscle activity in the FDI during trackpad usage is low compared to that in the bigger trapezius muscle during computer mouse usage [29,30]. As a result, it could be hard to rely on muscle tension and stiffness to infer stress using trackpad versus mouse data [4,27].

## Methods

### Subjects

We recruited 22 subjects (10 males [45%] and 12 females [55%]), with ages ranging from 19 to 68 (mean 37, SD 16), not screened for preferences in using a computer mouse or trackpad. We eliminated 4 subjects for whom our stressor did not have any effect on SRS, leaving a total of 18 subjects (8 males [44%] and 10 females [56%]). Of the remaining 18 subjects, 11 (61%) reported using a computer mouse and 9 (50%) reported using a laptop trackpad daily. The mean self-reported daily usage duration of the laptop trackpad (mean 2.6, SD 3.1 hours) was significantly lower (t_17_=2.1, *P*=.03) than that of the computer mouse (mean 5.2, SD 3.1 hours). Subjects provided written informed consent before participation and were given US $20 gift cards for their participation. The study was approved by the Institutional Review Board of Stanford University.

### Apparatus

**Figure 3:**
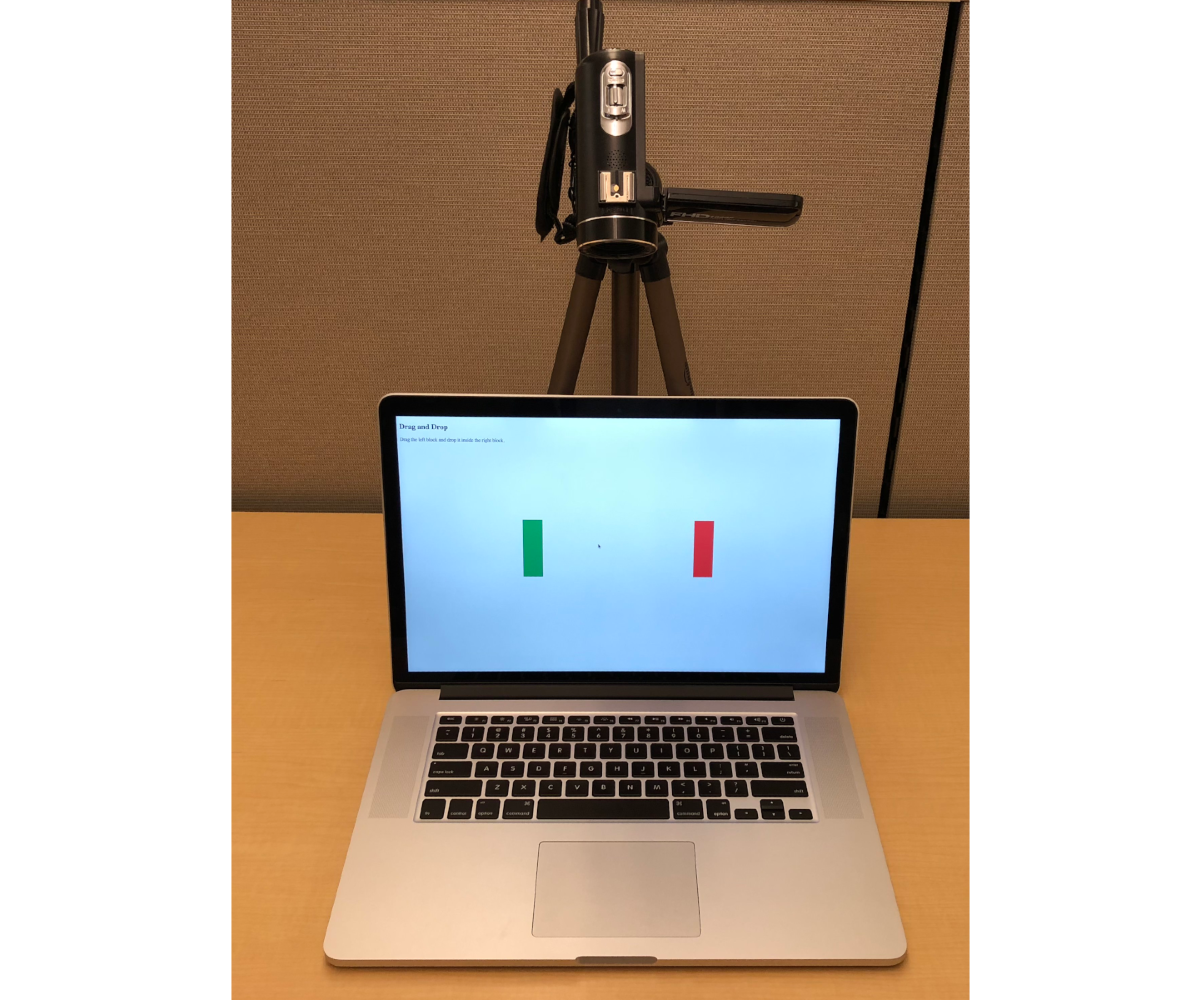
Apparatus: a 15-inch 2015 MacBook Pro (Apple Inc) with a passive movement logger teamed with an overhead camera for the ancillary recording of hand movement.

The experiment was performed in an office setting without any distractions. We used a 15-inch 2015 MacBook Pro (Apple Inc) (see Figure 3) equipped with a 140×70-mm Force Touch trackpad with sensitivity preset to a default value of 1.0. An overhead camera captured hand movement, and our logging software captured trackpad and cursor activity. The logger was implemented in C and Swift (Apple Inc) using Apple’s *MultiTouchSupport* framework. It recorded coordinates rounded to the nearest hundredth of a millimeter with an average sampling rate of 8.17 milliseconds (SD 3.75), multiple finger touches marked with ID numbers, contact shape under the finger (ie, major and minor axes of the ellipse), interaction type (ie, touching, dragging, etc), and pressed state (ie, active or inactive).

### Stimuli

The stimuli consisted of *Relaxation* and *Stressor* phases replicated from prior studies [4,5]. During the *Relaxation* phase, we instructed subjects to breathe deeply while viewing a soothing video, which is recommended over doing nothing [31]. The acute *Stressor* phase consisted of an arithmetic cognitive stressor combined with social evaluation (ie, the presence of an experimenter continuously observing and correcting if the subject made a mistake), derived from the Trier Social Stress Test [32], and enhanced with a biased financial stimulus. The test required subjects to perform a series of subtractions out loud (eg, subtract 13 from 2017, and so forth) under the scrutiny of the experimenter. If the subject made a mistake, or if he or she took more than four seconds to respond, the experimenter asked the subject to start again from 2017. The subject was offered an additional US $1 for every 10 consecutive arithmetic operations performed correctly, but only US $1 was to be deducted for each mistake. All subjects made mistakes that made them “lose” money, but after debriefing, they all received full compensation. The financial part of the stimulus based on performance could have been more frustrating than stressful for some subjects. However, it was added, as was done in our previous study [4], since frustration can further exacerbate acute stress.

### Experimental Tasks

**Figure 4:**
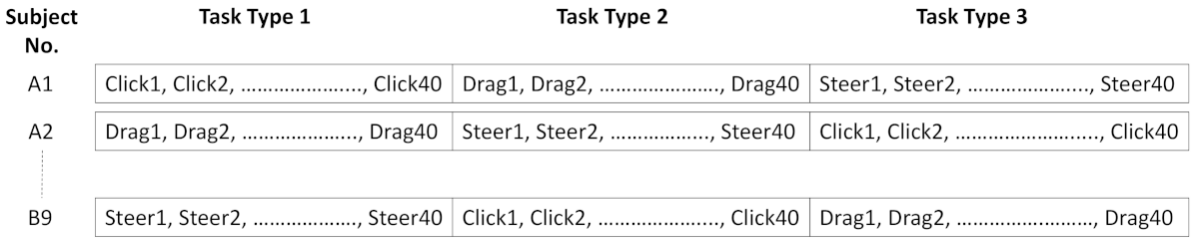
Example of distribution of different task types across different subjects.

All subjects performed three types of tasks—*click*, *steer,* and *drag and drop* (see Figure 1)—as in our previous study using the computer mouse [4]. Five different distance (D) and four different width (W) configurations were used for each of the three task types, resulting in a total of 20 configurations per task (see Table 1) [4]. Users performed two trials per configuration within each task type (see Figure 4). Tasks of the same type were grouped to prevent performance differences due to subjects needing to adjust to different task types. In total, each experimental condition, *tStressed* (stressed task) and *tRelaxed* (relaxed task), had 120 trials (3 task types × 40 trials).

### Experimental Design

#### Testing Procedure

**Figure 5:**
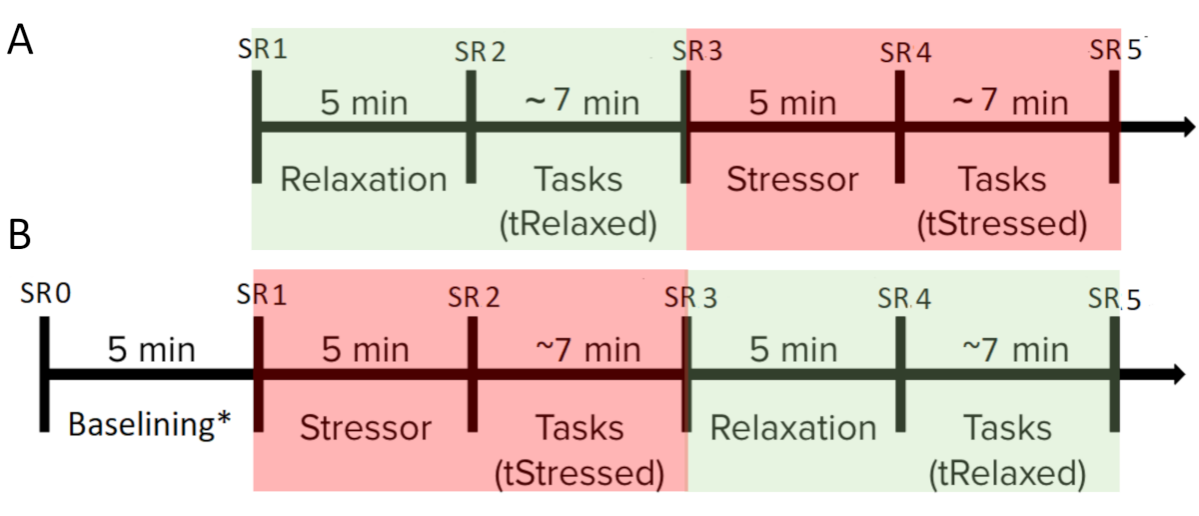
Study procedure with (A) *Relax-Stress* and (B) *Stress-Relax* counterbalanced arms. The *Relax* part is shown with a green transparent box and the *Stress* part is shown with a red transparent box. Data were collected at self-report marker (SR) time points 0-5. *For the *Stress-Relax* arm, before the stressor phase, a baselining phase was carried out to normalize the effects of external factors. *tRelaxed*: relaxed task; *tStressed*: stressed task.

The experiment consisted of four distinct phases (see Figure 5):

1. *Baselining* and *Relaxation*: a 5-minute phase designed to normalize the effects of any stressors, including any external factors, and to bring the subject to a baseline level.
2. *tRelaxed*: an approximately 7-minute phase (mean 6.8, SD 1.7 minutes) during which subjects were instructed to perform the randomized tasks—*click, drag and drop,* and *steer* (see Figure 1)—as quickly and accurately as possible.
3. *Stressor*: a 5-minute stress-inducing phase.
4. *tStressed*: an approximately 7-minute phase (mean 6.8, SD 1.5 minutes) during which subjects were instructed to perform the randomized tasks—*click, drag and drop,* and *steer* (see Figure 1)—as quickly and accurately as possible.

The 18 subjects were assigned to one of the two counterbalanced arms: *Relax-Stress* (3 males [17%] and 5 females [28%]) and *Stress-Relax* (5 males [28%] and 5 females [28%]) (see Figure 5). The *Stress-Relax* arm required an additional baselining phase, before *tRelaxed*, to normalize the effects of external factors before applying the stressor. Subjects wore an E4 wristband (Empatica Inc) to measure EDA in their nondominant hand and provided perceived self-reported *stress*, *tension,* and *concentration* levels on a scale of 0-10, before and after each phase (see Figure 5).

#### Data Processing

##### Self-Report and Ancillary Data

SRS was assessed using a simplified version of the PSS [11], an 11-point scale question—“What is your current level of stress?” where responses ranged from *Low* (0) to *High* (10)—immediately after completion of each phase (see Figure 5). To verify that the stressor did not induce cognitive alterations, we assessed perceived *concentration*, ranging from *Low* (0) to *High* (10), and measured *trial completion time*. Finally, we captured ancillary data on perceived muscle *tension*, ranging from *None* (0) to *High* (10). Self-report markers (SRs) in Figure 5 show the time points during the experiment where these data were collected. To estimate the self-reported metrics *during* any phase, the before and after values were averaged for that phase. For example, the average of SR4 and SR5 values for stress for subjects in arm A (see Figure 5) gave us an estimate of the SRS value *during* the *tStressed* phase.

All self-reported metrics were min-max normalized across the four phases—*Relaxation, Stressor, tRelaxed,* and *tStressed*—unless stated otherwise. For normally distributed data, where the Shapiro-Wilk test was not significant, we applied a 1-tailed paired *t* test. Otherwise, we applied a 1-tailed Wilcoxon signed-rank test. We used 1-sided comparisons, as we had prior knowledge about the expected direction of changes in the different metrics, and the stressor task we used has already been shown to be effective in inducing stress in prior studies [4,5,32]. Effect sizes (Cohen *d*) are provided for within-group changes for primary measures, where Cohen *d* values between 0.20 and 0.49 indicate a small effect, values between 0.50 and 0.79 indicate a medium effect, and values of 0.80 and above indicate a large effect [33]. For any bivariate correlations, we used the Pearson correlation coefficient for normally distributed data; otherwise, we used Spearman rho.

Out of 22 subjects, 4 (18%) showed no change in SRS across the two extreme phases—*Stressor* and *Relaxation*—and were excluded from all analyses, leaving a working set of 18 subjects.

##### EDA Processing

We eliminated 3 of the 18 subjects (17%) before the analysis, leaving a total of 15 subjects (83%) for EDA analysis: 1 subject did not have a signal due to loose contact, another had an anomalous signal above 20 µS [34], and 1 had more than 5% noisy 5-second epochs estimated with the *EDA Explorer* [35]. For the remaining 15 subjects, EDA signals were filtered using a 6th-order 1-Hz low-pass Butterworth filter [36]. Then, using Ledalab, version 3.2.5 [37], we obtained the tonic mean, phasic mean, and the number of SCR peaks with an amplitude above 0.01 µS. Only data from *tRelaxed* and *tStressed* phases were considered for the EDA analyses.

##### RQ1: MSD Model Evaluation

**Figure 6:**
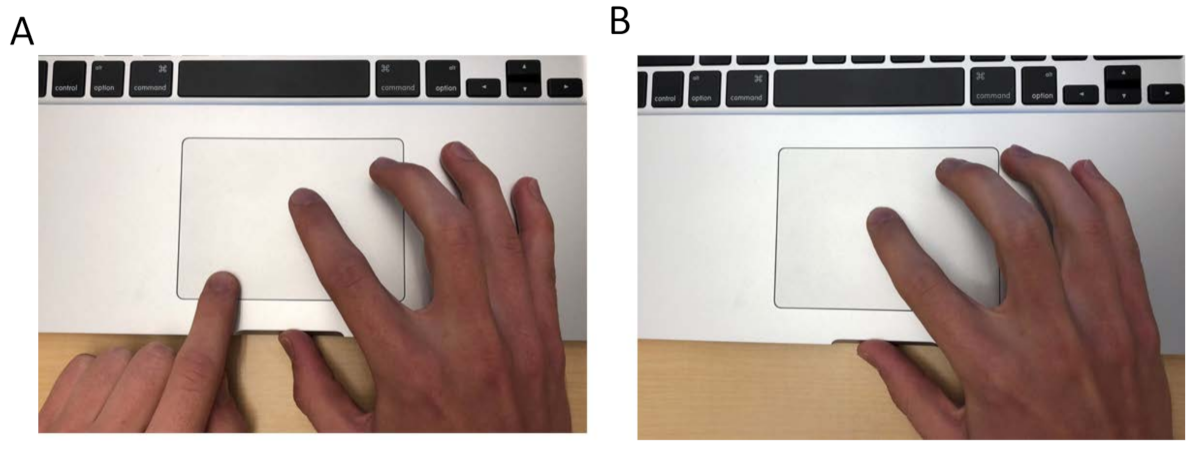
(A) multiple hands or (B) multiple fingers being used while interacting with the trackpad.

**Figure 7:**
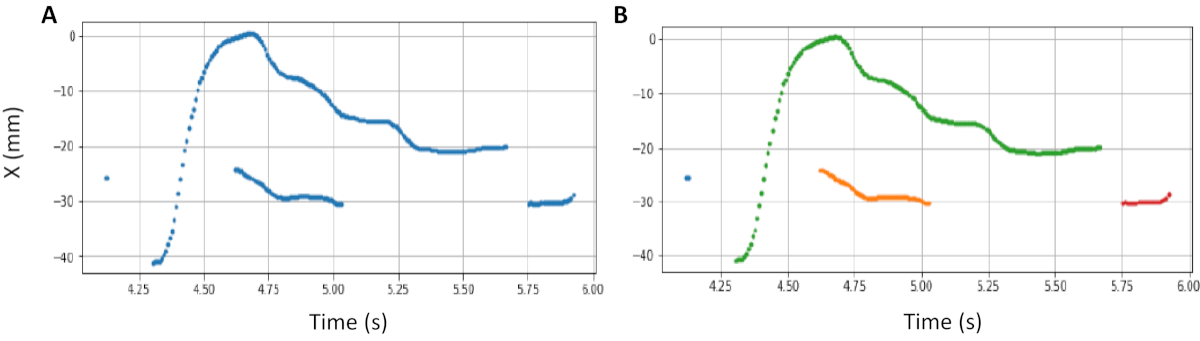
Example traces from a *click* trial. (A) Multiple traces at the same time (see between 4.6 and 5.0 seconds) suggests the use of multiple fingers from the same or different hands at the same time. The gap between 5.70 and 5.75 seconds indicates that the subject lifted his or her finger and then placed the same or another finger back. (B) Identifying the largest stroke (green trace) and other ancillary strokes due to the use of different fingers (orange and red traces). X represents the horizontal movement on the trackpad.

We modeled trackpad finger trajectories using a linear predictive coding (LPC) inverse filtering technique [38] as in Sun et al, who successfully modeled the arm movement as an approximation of a step response of a single-degree-of-freedom MSD system [4]. The LPC approximation for the MSD model has been used in several other studies, like in our previous work using steering wheel data [5] and also by Guo et al [39], using data from touchpad interactions. However, unlike mouse movements, which are primarily continuous and more standardized across subjects, there are many quirks in the way different individuals interact with the trackpad. Since subjects were given the freedom to use the trackpad as normally as possible, we observed that 17 out of 18 subjects (94%) used multiple fingers from their dominant hand, and/or used fingers from both hands (see Figure 6), creating many discontinuities in the finger strokes during each trial (see Figure 7). Across all subjects, a mean of 38.0% (SD 13.9) of the trials had at least one gap in the continuity of the finger stroke because subjects either used multiple hands (see Figure 6, A), multiple fingers (see Figure 6, B), or even the same finger, but the finger hovered on the trackpad. We used our ancillary videos to observe this behavior, as it was impossible to use logger data alone to verify that the subject used the same finger multiple times or separate fingers during one trial.

We identify gaps (see Figure 7, A) by searching for large variations in x or y coordinates and keeping points that represented the largest continuous movement of the underlying stroke (see Figure 7, B—green trace). We fitted an approximation to the MSD model [4] to this stroke and extracted Γ and ω for the horizontal x-direction, which contained most of the displacement information for our tasks.

##### RQ2: Measurements of Finger Dynamics From Trackpad Interactions

Under stress, wrist and forearm muscles present changes in mean force production [19] and motor control variability [17]. Therefore, we measured mean and SD values for three typical finger dynamics measurements: velocity, acceleration, and contact area under the finger. We kept all strokes and substrokes for every trial by identifying the gaps between partial movements (see Figure 7, B) and averaged their metrics for each trial. We estimated the contact area of the ellipse (*π* × a × b) between the finger and the trackpad using the major axis (a) and minor axis (b) provided by the logger. We calculated finger velocity by differentiating position information and finger acceleration by differentiating velocity information. We used only horizontal (x-direction) displacement data, which contained most of the information for our tasks.

### Statistical Modeling and Sensitivity Analysis

First, we evaluated a *Mixed Task* omnibus model independent of specific task types—*click*, *drag and drop,* and *steer*—and task configurations (W × D). Values for each task type were obtained by averaging across all configurations and repetitions (ie, 40 trials) for each of the *tStressed* and *tRelaxed* phases for each subject. Then, a single value for each *tStressed* and *tRelaxed* phase was obtained after averaging across all three task types for each subject. Variables with significant differences across the *tStressed* and *tRelaxed* phases in the *Mixed Task* model were further examined using *Task-Specific* models. To avoid inflation, we applied Bonferroni correction (0.05/3 = 0.017) for *Task-Specific* models. Finally, for the type of task with the most significant difference, we applied a sensitivity analysis comparing the *initial* 10% (ie, 4 trials out of 40) of data across the *tStressed* and *tRelaxed* phases. We also carried out post hoc power (*β*) analyses on all finger dynamics measures that were found to be significant. Additionally, to check if the order of stress-relax condition has any effect, we investigated the *Order* (ie, A vs B; see Figure 5) × *Condition* (ie, stress vs relax) interaction effect using analyses of variance on all finger dynamics measures that were found to be significant.

## Results

### Mental Stress Validation

#### SRS and Tension Metrics

**Figure 8:**
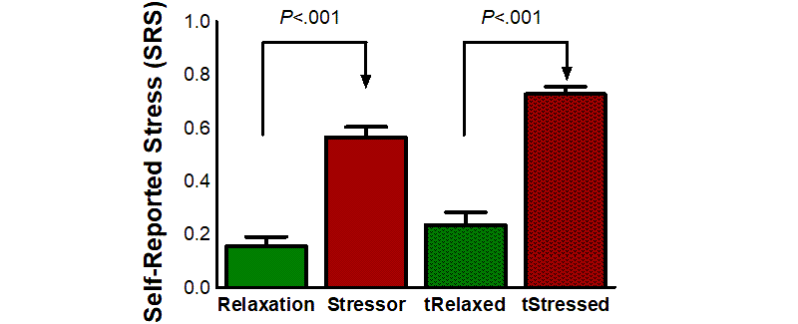
Levels of min-max normalized self-reported stress. Error bars represent SE. *tSRelaxed*: relaxed task; *tStressed*: stressed task.

On average, the SRS was significantly higher *during* the *Stressor* phase (mean 0.56, SE 0.04) compared to the *Relaxation* phase (mean 0.15, SE 0.03) (*Z*_17_=3.72, *P*<.001). The SRS was also significantly higher *during* the *tStressed* phase (mean 0.72, SE 0.03) compared to the *tRelaxed* phase (mean 0.23, SE 0.04) (t_17_=13.06, *P*<.001). Figure 8 shows a comparison of min-max normalized SRS values for the four phases of the experiment. Table 2 shows the average raw SRS values at the end of the *Relaxation, tRelaxed, Stressor,* and *tStressed* phases. It can be observed that SRS levels go down over time during the *tStressed phase*.

**Table 2:** Average raw self-reported stress values across all subjects at the end of different phases.

Time point	Mean (SE)
End of *Relaxation* phase	2.27 (0.42)
End of *tRelaxed*^a^ phase	3.78 (0.42)
End of *Stressor* phase	6.22 (0.61)
End of *tStressed*^b^ phase	4.50 (0.42)

^a^*tRelaxed*: relaxed task.

^b^*tStressed*: stressed task.

In addition to SRS, we found that the perceived *tension* was significantly higher *during Stressor* (mean 0.52, SE 0.05) versus *Relaxation* (mean 0.09, SE 0.02) (*Z*_17_=3.59, *P*<.001) phases and *during tStressed* (mean 0.78, SE 0.03) versus *tRelaxed* (mean 0.29, SE 0.04) (t_17_=11.79, *P*<.001) phases. Furthermore, perceived *tension* and *SRS* were highly correlated (*r*=.74, *P*<.001).

No statistical differences (t_17_=0.58, *P*=.29) were observed for *trial completion time* between *tRelaxed* (mean 2.04, SE 0.14 seconds) and *tStressed* (mean 2.10, SE 0.13 seconds). Additionally, we found no statistically significant differences for perceived *concentration during Stressor* versus *Relaxation* phases (*Z*_17_=1.46, *P*=.07)*,* and *tStressed* versus *tRelaxed* phases (*Z*_17_=0.56, *P*=.28). This shows that our stressor elicited an affective response as opposed to a response due to changes in cognitive performance.

#### Physiological Stress

All metrics for EDA were significantly higher. Mean phasic EDA *during tStressed* (mean 0.040, SE 0.015 S) compared to *tRelaxed* (mean 0.020, SE 0.006 S) was higher (*Z*_14_=2.33, *P*<.01); mean tonic EDA *during tStressed* (mean 4.223, SE 1.389 S) compared to *tRelaxed* (mean 2.057, SE 0.806 S) was higher (*Z*_14_=3.181, *P*<.001); and the number of EDA *peaks* (ie, SCR events) *during tStressed* (mean 68.2, SE 16.9) compared to *tRelaxed* (mean 40.4, SE 11.7) was higher as well (*Z*_14_=2.86, *P*<.01).

Overall, both SRS and EDA evaluations showed that our stressor was effective in inducing acute stress, as planned.

### Trackpad Interactions

#### Mixed Task Models

Both the mean and SD of contact area under the finger were significantly different between *tRelaxed* and *tStressed* phases with large effect sizes (Cohen *d*>0.8) and large post hoc power (*β*>97%) (see Table 3). The mean and SD of velocity and acceleration, as well as the two LPC-approximated biomechanical MSD parameters (Γ and ω), were not significantly different. Paired *t* tests were carried out for all these measures except for Γ, for which a Wilcoxon signed-rank test was carried out. For mean contact area, the *Order* × *Condition* interaction effect was not significant (*F*_1,32_=1.87, *P*=.18). However, for the SD of contact area, the interaction effect was significant (*F*_1,32_=11.17, *P*<.01), suggesting that the *Order* of the conditions did have a significant effect on the overall SD of the contact area across the two *Conditions*.

**Table 3:** Summary of descriptive statistics between tRelaxed and tStressed phases for the Mixed Task model.

Measure		*tRelaxed*^a^, mean (SE)	*tStressed*^b^, mean (SE)	*t* test (17)	*Z* test (17)	*P* value	Cohen *d*
**Normalized velocity**							
	Mean	0.47 (0.16)	0.49 (0.12)	0.50	N/A^c^	.31	0.15
	SD	0.39 (0.14)	0.47 (0.20)	1.42	N/A	.09	0.48
**Normalized acceleration**							
	Mean	0.35 (0.15)	0.43 (0.18)	1.36	N/A	.10	0.49
	SD	0.31 (0.17)	0.42 (0.17)	1.54	N/A	.07	0.64
**Normalized area**							
	Mean	0.40 (0.18)	0.55 (0.16)	2.18	N/A	*.02* ^d^	0.87
	SD	0.39 (0.17)	0.53 (0.13)	2.08	N/A	*.03*	0.92
**Mass-spring-damper model**							
	Γ (damping ratio)	0.538 (0.007)	0.540 (0.006)	N/A	0.11	.46	0.09
	ω (damping frequency) (rad/s)	0.256 (0.006)	0.261 (0.005)	1.21	N/A	.12	0.17

^a^*tRelaxed*: relaxed task.

^b^*tStressed*: stressed task.

^c^N/A: not applicable.

^d^Italicized values indicate significance (*P*<.05).

#### Task-Specific Models

**Figure 9:**
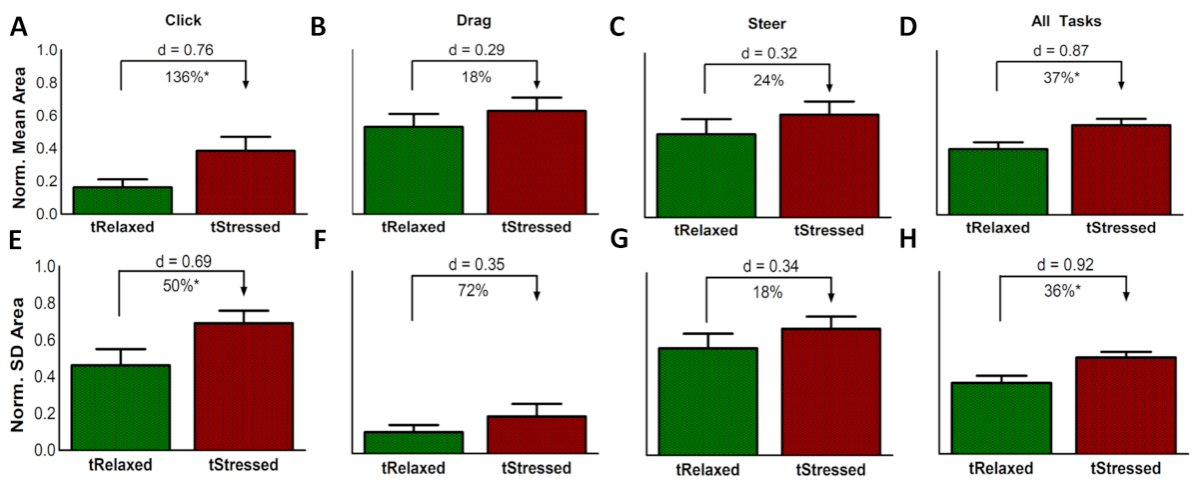
Bar plots (Mean ± SE), effect size (Cohen *d*), and the percentage difference between *tRelaxed* (relaxed task) and *tStressed* (stressed task) phases of (A, E) *click*, (B, F) *drag*, (C, G) *steer* tasks, and overall combining (D, H) *All Tasks* (*Mixed Task* model) for both mean and SD of contact area, respectively. (*) for click indicates *P*<.05/3 = .017 (Bonferroni correction), and (*) for *All Tasks* indicates *P*<.05.

*Task-Specific* models for mean and SD of the contact area under the finger (see Figure 9) were performed for all three task types. Significant differences (*P*<.02) between *tStressed* and *tRelaxed* were observed for *click* tasks only, both for mean area (*Z*_17_=2.37, *P*=.009, *β*=91%) and for SD of the area (*Z*_17_=2.33, *P*=.01, *β*=86%). Overall, *click* tasks (see Figure 9, A and E) show large percentage differences between *tRelaxed* and *tStressed* phases.

The interaction effects for mean and SD of contact area during *click* tasks were not significant (mean area: *F*_1,32_=0.00, *P=*.98; SD area: *F*_1,32_=1.39, *P=*.25). We also evaluated the interaction effects for SD of the contact area for *drag and drop* (*F*_1,32_=1.82, *P=*.19) and *steer* (*F*_1,32_=4.48, *P=*.04)*,* the latter of which was significant, suggesting that the significant interaction in SD of the contact area variable in the *Mixed Task* model (see the Mixed Task Models section) is mainly due to significant interaction effect only in *steer* task trials. Overall, our results suggest that finger dynamics from *click* tasks alone, which are the most common computer interactions (see Discussion section), and are unaffected by *Order* effects, can be used as robust measurements to detect acute stress.

### Sensitivity Analyses

#### Data Reduction

In this section, we focus on *click* data, which was the only task type contributing to the overall differences in the *Mixed Task* model. We started looking for data closest to the stressor (ie, the *initial* 10% [4 *clicks* out of 40]), and we intended to explore bigger segments (ie, 20%, 30%, etc) in case we did not find a signal sufficient to detect acute stress across the two conditions. To our surprise, with only 4 *clicks*, we could see a significant difference across the two phases (see Table 4). This result may be because the effect of the stressor is higher earlier during the *tStressed* phase, and it decays over time (see Table 2). Similar sensitivity results have been observed in other biomechanical models of stress [5]. Neither of the two variables mentioned in Table 4 showed any significant interaction effect (mean area: *F*_1,32_=0.00, *P=*.99; SD area: *F*_1,32_=0.50, *P=*.49), which reconfirms that *click* tasks are not influenced by *Order* effects.

**Table 4:** Summary of descriptive statistics between tRelaxed and tStressed phases pertaining to only the initial 10% of click trials.

Measure	No. of *clicks* (N=40), n (%)	*tRelaxed*^a,^ mean (SE)	*tStressed*^b^, mean (SE)	*t* test (17)	*P* value	Cohen *d*	*β*, %
Mean area (mm^2^)	4 (10)	270.9 (6.3)	284.6 (8.1)	2.81	*<.01* ^c^	0.44	56
SD area (mm^2^)	4 (10)	16.7 (1.6)	21.2 (1.3)	2.88	*<.01*	0.73	91

^a^*tRelaxed*: relaxed task.

^b^*tStressed*: stressed task.

^c^Italicized values indicate significance (*P*<.05).

#### Click Rate Manipulation

Our original manipulation generated *click* trials at a rate of roughly 1 *click* per 3.5 seconds: 40 *click* trials were completed in an average of 136 seconds (SD 31). To create a scenario in which the *clicks* are obtained at a slower rate, which could also be plausible in the wild, we tested the artificially slowest rate by decimating the samples, using only 10% of the data distributed across the entire range of the production of the *click* trials. We picked the first, the last, and 2 random *click* trials from 1 to 40, in this way simulating a scenario in which *clicks* are generated once every ~45 seconds. As it turns out, both area measures were still significantly different across two stress conditions. The mean of the mean area for *tStressed* was 277 mm^2^ (SE 8.61) and for *tRelaxed* was 267 mm^2^ (SE 6.4) (t_17_=2.07, *P=*.03, *d*=0.32 , *β*=37%). The mean of the SD area for *tStressed* was 22 mm^2^ (SE 1.8) and for *tRelaxed* was 18 mm^2^ (SE 1.5) (t_17_=1.86, *P*=.04, *d*=0.54, *β*=71%). Further, no significant interaction effect was found for either of these two measures as well (mean area: *F*_1,32_=0.14, *P=*.72; SD area: *F*_1,32_=1.98, *P=*.17).

#### Individual Differences

**Figure 10:**
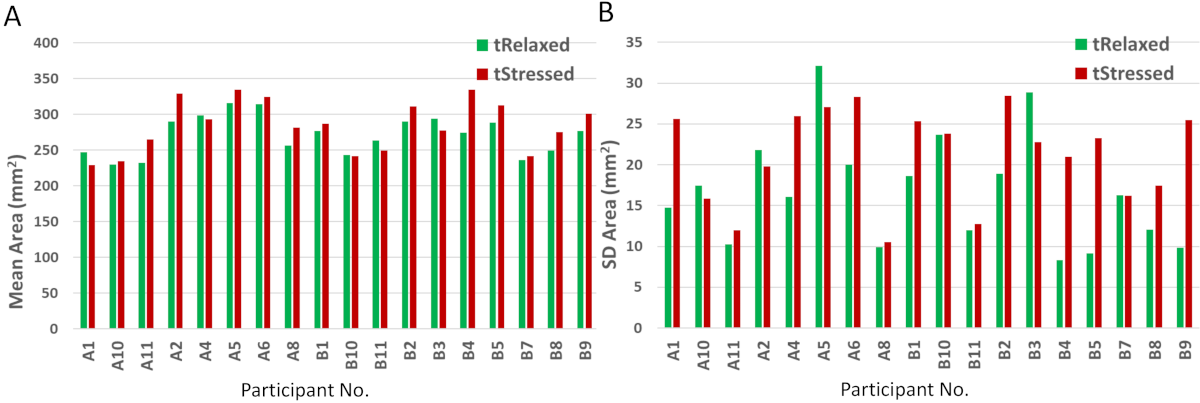
Individual differences for (A) mean and (B) SD of the contact area under the finger across the initial 10% of the *click* trials. *tRelaxed*: relaxed task; *tStressed*: stressed task.

When looking at individual differences for all tasks, 12 out of 18 (67%) participants showed an expected increase in either the mean or the SD of the area under the finger. For the initial 10% (ie, 4 out of 40 *clicks*), 13 out of 18 (72%) subjects had changes in the expected direction for either mean or SD (see Figure 10). Participants A1, A4, B10, B1, and B3 showed an opposite trend for the mean area (see Figure 10, A), while participants A10, A2, A5, B3, and B7 showed an opposite trend for SD area (see Figure 10, B). Subject B3 showed an opposite trend for both measures. If we were to consider a hard evaluation where both metrics must occur at the same time, we would see 11 out of 18 (61%) subjects satisfying the expected change. However, if we were to detect stress whenever either of the two measures has the right trend, we would detect stress changes in 17 out of 18 (94%) subjects. This latter approach is plausible, as both measurements are theoretically linked to changes in either muscle tension or motor control alterations due to stress (see section RQ2: Measurements of Finger Dynamics From Trackpad Interactions).

## Discussion

### Principal Findings

Enabling precision health and effective stress management regimes are dependent on the evolution of ubiquitous real-time stress sensors and algorithms. An ideal scenario would be to passively obtain data from everyday use devices, rather than having people wear sensors or answer survey questions. In this study, we aim at assessing the feasibility of using passively obtained laptop trackpad data to detect acute stress. We present evidence that binary levels of acute stress (ie, whether it is present or not) can be detected from finger strokes alone.

In response to RQ1, we found out that our approximation to an MSD model, previously used with data from the computer mouse [4], cannot be used directly on trackpad data. Our analyses provide new insights into the complexity of the use of finger stroke data from trackpad operations for applying an MSD model. Unlike a computer mouse, handling a laptop trackpad is subject dependent. Some people may employ multiple hands and/or multiple fingers to perform the same tasks. This creates challenges to process the data due to the gaps present in the strokes and the difficulty of obtaining a clean intentional segment of data, with a clear *beginning* and *end*, required to get meaningful MSD results. These limitations leave little useful data for analysis, and we did not observe a difference in either of the two MSD parameters (ω and Γ) between the *tRelaxed* versus *tStressed* phases. However, our analyses were restricted to the largest stroke per trial. It is possible that in the wild, where we can collect larger amounts of data that would allow us to extract valid displacement segments, an MSD approach may still be useful to assess changes due to muscle tension under acute stress.

Our RQ2’s results are encouraging, showing the robustness of the mean and SD of the finger contact area as a stress sensor. Both metrics were higher during the *stressed* tasks as compared to the *relaxed* tasks. We show that as few as 4 *click* trials per subject are sufficient to see a significant difference, which is promising for longitudinal studies. Overall, we present evidence of the use of the finger contact area from laptop trackpad measurements to detect acute stress while performing common computer tasks.

### Acute Stress Detected Using Click Finger Dynamics

This is the first study showing that the mean and SD of the contact area under the finger on a trackpad are higher under stress compared to a relaxed state (ie, *Mixed Task* model). Theoretically, the mean value is related to mean force production [19] and higher variability [17] under stressful situations in the wrist and forearm muscles. There may be other underlying mechanisms (eg, changes in hand or body posture [40]) through which stress may influence finger dynamics, and future studies should monitor those variables as well. In the *Task-Specific* model, differences in mean and SD of the contact area under the finger were different for *click* trials. If combined, mean *or* SD, we could detect up to 94% of acute stress events. This is encouraging because *clicking* tasks represent nearly 70% of all computer mouse events in a typical day [41].

With just the initial 10% of *clicking* trials (ie, 4 *clicks* out of 40), we could still see small and medium effect size differences in mean and SD of the contact area under the finger. Since *click* trials were not always carried out right after the stressor, the difference is not due to ordering effects; here we are referring to ordering of *click, steer,* and *drag and drop,* and not the ordering of *stress-relax* conditions. We attribute the strength of using so little data to the proximity to the stressor, as there is a clear decay of stress over time, which would clearly affect values averaged over longer periods. Furthermore, we simulated a scenario where *clicks* are produced at a different rate (ie, going from 1 *click* produced every ~3.5 seconds to every ~45 seconds). We obtained significant results even for the lowest *click* productivity we could simulate with our data (1 *click*/45 s). In a separate pilot study, collecting computer mouse data “in the wild,” we have seen that a typical user may generate a *click* every 10-30 seconds, and at least 1000 *clicks* are generated in any given working day. Thus, we feel confident we can use our findings as the basis for a passive, continuous, scalable, inexpensive, and unobtrusive stress sensor. In the wild, of course, changes in muscle tension would not only be due to affective (ie, ANS) processes but also due to cognitive processes (see Potential Interaction Effects With Task Performance section). Ultimately, a reliable “in the wild” stress detection system would have to combine multiple modalities, such as biomechanical, behavioral, and physiological sensors, as well as contextual information.

### Potential Interaction Effects With Task Performance

Mean and SD of finger velocity and acceleration were similar across the two phases, as well as *trial completion time*. This reconfirms that our stressor did not change the cognitive performance of the subjects but only their affective state. However, the relationships between stress, velocity, and acceleration may not be simple. For example, one may say that under a stressful situation, a user may want to move the fingers with higher velocity and/or acceleration; however, after a certain point, it will also start affecting the accuracy of this fine motor control task [42]. Thus, the user may not use higher velocities and/or acceleration under stress. Further studies with different elicitations to modify the performance rewards must be performed to observe interaction effects between these variables.

### Contrasting With Other Touch Technologies

At least two studies have shown higher finger pressure under stress [28] and under frustrating [7] conditions while using touchscreen devices. However, the usage and handling of touchscreen devices are not the same as that of a trackpad, in terms of fine and precise interaction [43]. The capacitive technologies used in touchscreens and trackpads are different in terms of how much pressure needs to be applied: typically, the trackpad requires stronger pressure [43]. Additional research would be needed to determine if our finger dynamics from contact area (ie, mean and SD) can be translated to other types of touch-sensitive devices.

### Limitations

Our study has four limitations related to sample size, stressor effect size, apparatus, and assumptions for modeling. First, because of the small sample size (N=18) we could not investigate the effects of age, gender, length of experience with trackpads, and frequency of day-to-day usage of trackpads. We also could not build reliable predictive models (eg, using different ML techniques), which could parse out the importance of various features in predicting acute stress. Second, given the mild nature of the stressor, not all users (18/22, 82%) responded to our manipulation. Nevertheless, this level of efficacy is expected for a mild stressor and in line with our prior study [6]. Third, although we do not expect the behavior of users to be fundamentally different across different laptops, our experiment should be revalidated on other models of trackpads. Fourth, using finger contact area as a proxy to pressure assumes a linear relationship between the two. However, factors like the angle of the finger can also influence this relationship. Since the time of the data collection for this study, Apple has updated the *MultiTouchSupport* framework, and it now also provides information about pressure and capacitance density underneath the fingers. Additional models collecting this new data would be quite useful to strengthen the precision of our detection method further.

### Future Directions

We plan to expand current results by running lab experiments with larger sample sizes; by logging other actions performed using trackpads, such as scrolling and multiple finger gestures; and by collecting other behavioral metrics, such as *click* production rate, the timing between *clicks*, etc. Once we collect more data, both in the laboratory settings and “in the wild,” we aim at building predictive models that include both biomechanical and behavioral metrics to increase the accuracy and specificity of stress prediction in real time.

As we move out of the lab, we will explore ways to carry out longitudinal studies collecting data from our passive loggers across devices (ie, mice, trackpads, touchpads), contextual data, and stress “labels” obtained from empirical sampling methods or physiological measurements. As we accumulate richer and bigger datasets, we plan to investigate data-intensive unsupervised and supervised ML methods to optimize data collection, preprocessing, and prediction. For example, we plan to investigate algorithms to parse types of events and movements (eg, *clicks*, *drag and drop*, etc) or to select segments that may have the right morphology to derive biomechanical features (eg, MSD parameters, contact area, etc). Ultimately, we hope that our lab and field findings can help select the appropriate behavioral, biomechanical, contextual, and perceptual features (ie, feature engineering) to train ML systems to be able to detect stress levels throughout the day.

### Conclusions

The current methods for sensing stress are often inconvenient, expensive, or suffer from limited adherence. In this lab study, we show the efficacy of repurposing signals from a laptop trackpad to detect acute mental stress. We showed that with a handful of *click* events, we could use the mean and SD of the finger contact area to obtain a binary estimation of acute stress (ie, whether it is present or not). We validated our sensing models against well-known stress metrics such as self-reports and EDA. Complementarily, we validated that we did not elicit performance or cognitive confounds that may affect our affective stress elicitation. These results provide a firm baseline toward our future goal of deploying our algorithms in the wild, leveraging the trackpad as a potential unobtrusive, scalable, continuous, and inexpensive stress sensor.

## Abbreviations

ANS: autonomic nervous system
β: post hoc power
D: distance
ECG: electrocardiogram
ED: extensor digitorum
EDA: electrodermal activity
EMG: electromyography
ESM: empirical sampling methods
FDI: first dorsal interosseous
Γ: damping ratio
HRV: heart rate variability
LPC: linear predictive coding
ML: machine learning
MSD: mass spring damper
ω: damping frequency
PCD: personal computing device
PSS: Perceived Stress Scale
RQ: research question
SCR: skin conductance response
SNS: sympathetic nervous system
SR: self-report marker
SRS: self-reported stress
tRelaxed: relaxed task
tStressed: stressed task
W: width
